# Protein kinase A is a target for aging and the aging heart

**DOI:** 10.18632/aging.100138

**Published:** 2010-04-25

**Authors:** Linda C Enns, Christina Pettan-Brewer, Warren Ladiges

**Affiliations:** Department of Comparative Medicine, School of Medicine, University of Washington, Seattle, WA 98195, USA

**Keywords:** lifespan extension, obesity resistance, enhanced cardiac function, mouse models of aging, AMPK, beta adrenergic receptors, leptin signaling

## Abstract

PKA is an important mediator of signal transduction downstream of G-protein-coupled receptors and plays a key role in the regulation of metabolism and triglyceride storage. It is a ubiquitous cellular kinase that phosphorylates serine and threonine residues in response to cAMP. PKA consists of two regulatory subunits, RI and RII, that are activated by cAMP to release two catalytic subunits, Cα and Cβ. We have shown that C57/BL6J male mice lacking the regulatory RIIβ subunit have extended lifespan and are resistant to age-related conditions including cardiac decline. In addition to being protected from diet-induced pathologies, PKA Cβ null mutant mice are protected from age-related problems such as weight gain and enlarged livers, as well as cardiac dysfunction and hypertrophy.  Several possible mechanisms for the age sparing effects of PKA inhibition are discussed including A kinase anchoring protein signaling, alterations in the β-adrenergic pathway, and activation of AMPK.  Since PKA is a major metabolic regulator of gene signaling, the human gene homologs are potential pharmacological targets for age-related conditions including heart disease associated with declining cardiac performance.

## Loss of function of Protein kinase A (PKA) mediates anti-aging effects

**Figure 1.**
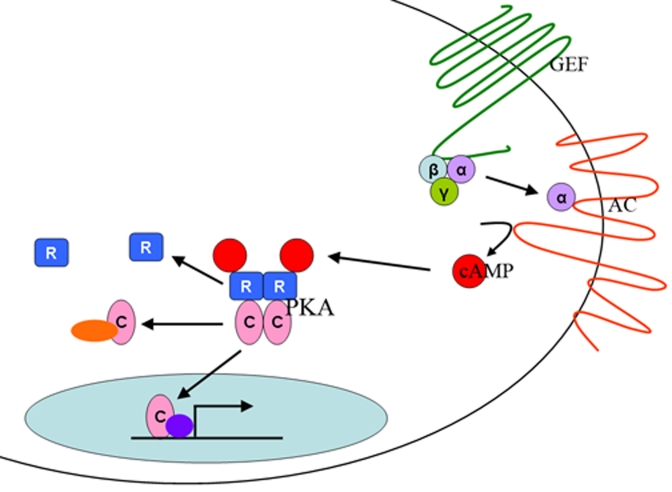
**The PKA pathway.** The PKA pathway is a nutrient sensing pathway.  In mammals, nutrients are sensed by a G-protein (GEF) that activates an adenylyl cylase (AC).  AC produces cAMP, which binds to the regulatory subunits (R) of the PKA holoenzyme, releasing the catalytic subunits (C), which are then free to enter the nucleus of the cell and activate gene transcription or to interact with other signaling proteins in the cell.

PKA is an important mediator of signal transduction downstream of G-protein-coupled receptors and plays a key role in the regulation of metabolism and triglyceride storage. It is a ubiquitous cellular kinase that phosphorylates serine and threonine residues in response to cAMP [1]. PKA is dependent upon cAMP for functional activation.  Adenyl cyclase (AC) is an upstream regulator of cAMP and PKA.  PKA consists of two regulatory subunits and two catalytic subunits (Figure 1).  cAMP binds to the regulatory subunits, releasing the catalytic subunits which are then free to interact with and phosphorylate downstream targets.  There are four isoforms of the regulatory subunit (RIα, RIβ, RIIα, RIIβ) and three types of catalytic subunits (Cα, Cβ, Cγ), each of which demonstrates different patterns of tissue expression and subcellular localization [2,3]. Our published studies have shown that C57/BL6J male mice lacking the regulatory RIIβ subunit have extended lifespan and are also resistant to age-related conditions including cardiac decline [4], summarized in Table 1. There was no lifespan advantage seen in PKA RIIβ females. Young RIIβ null and WT littermates weigh about the same and have about the same amount of body fat and lean body mass, but with age, there is a striking difference in these parameters between the two genotypes for either gender. Both genotypes eat about the same amount, so the body composition differences cannot be attributed to differences in food intake.  RIIβ null males are more insulin sensitive that WT littermates, regardless of age, but old (17 mos) RIIβ null females are also extremely insulin sensitive compared to WT littermates, suggesting that this is not the physiological factor responsible for the longevity phenotype observed exclusively in males.

In contrast, our data has shown that body composition, including body weight, percent body fat mass, and percent lean body mass, are correlated with lifespan in WT C57/BL6J male, but not female mice [4] suggesting that body composition may be a physiological factor contributing to the difference in lifespan phenotypes between mutant males and females.

## Cardiac aging is delayed in PKA RIIβ null mutant mice

 Echocardiographic parameters in mice, particularly left ventricular hypertrophy, diastolic dysfunction and impaired myocardial performance index, show progressive and highly reproducible changes with advancing age which parallel those of human aging [5]. Aging is accompanied by slowly progressive and irreversible structural changes and functional declines in the heart. Echocardiography in healthy populations from the Framingham Study and the Baltimore Longitudinal Study on Aging showed an age-dependent increase in the prevalence of left ventricular hypertrophy, a decline in diastolic function, and relatively preserved systolic function at rest but a decline in exercise capacity, as well as an increase in the prevalence of atrial fibrillation (reviewed in [6]). Diastolic heart failure, defined as symptoms of heart failure in the setting of diminished diastolic function, is pervasive in older individuals and markedly increases the risk of mortality [7]. Greater than half of individuals over the age of 75 with validated congestive heart failure had diastolic dysfunction and in many individuals this was clinically unrecognized and untreated. Diastolic dysfunction is also a major contributor to exercise intolerance in the elderly population. An age-dependent impairment of myocardial performance index (MPI) has also been shown [8].

Using echocardiography, we have seen cardiac dysfunction as early as 10-12 months of age in wild type C57BL/6 mice, which continually progresses with increasing age. Heart weights of young PKA RIIβ mutant mice are similar to WT littermates, but at 24 months of age, mutants showed significantly lower left ventricular masses compared to WT mice [4].  Doppler imaging on these older mice, employed to measure the velocity of the mitral valve annulus,  isovolumic contraction and relaxation times and ejection times, also showed superior Ea/Aa ratios in the mutants, indicating a resistance to age-related diastolic dysfunction, and a lower average myocardial performance index (MPI) indicative of superior global ventricular function [8]. These observations indicate a cardiac protective affect of the RIIβ deletion and suggest a possible connection with the Yan et al. findings [9]  that the absence of AC5 is protective of cardiac function.  The fact that RIIβ is not expressed, or expressed at very low levels in cardiac tissue suggests that signaling from adipose tissue or the brain may be involved.  It is also possible that the delayed cardiac function is a secondary effect due to lack of adiposity.

**Table 1.** Summary of aging phenotypes in end of life RIIbeta nulle males.

**Phenotype**	**Males**	**Females**
Lifespan	Extended	No extension
Body fat gain	Suppressed	Suppressed
Insulin resistance	Suppressed	Suppressed
Cardiac dysfunction	Suppressed	To be determined
Cardiac hypertrophy	Suppressed	Suppressed

PKA plays multiple roles in heart function. Its phosphorylation in the cardiac myocyte regulates many processes including contraction, metabolism, ion fluxes, and the transcription of many different genes [10]. Altered PKA signaling has been implicated in a number of physiological problems leading to cardiomyopathy.  For example, the onset of cardiac hypertrophy is influenced by alterations in muscle-specific A-kinase Anchoring Protein (mAKAP) signaling in myocytes. AKAPs bind to PKA regulatory subunits such as RIIβ, in order to subcellularly localize and modulate interactions between PKA and its downstream targets [11].  PKA is also involved in the downstream regulation of the β-adrenergic pathway. Stimulation of β-adrenergic receptors (β-ARs) in the heart leads to the PKA-dependent phosphorylation of multiple intracellular targets in cardiac myocytes including the L-type Ca2^+^ channel in the sarcolemma, the ryanodine receptor (RyR2), and phospholamban in the sarcoplasmic reticulum [12, 13]. Deficiencies in this pathway have been linked to increased baseline myofibrillar Ca2+ sensitivity and subsequent cardio-myopathy in humans, due to reduced phosphorylation of downstream targets such as cardiac troponin I [14]. The β-adrenergic pathway is known to be enhanced in RIIβ null mice [15], which could help provide a possible mechanism for the cardiac sparing effects in seen in these mice.  Paradoxically, activated βAR signaling has also been implicated in the failing heart.  Chronic heart failure is associated with an increase in circulating catecholamines [16], PKA phosphorylation of RyR2 is markedly increased in failing human hearts [17], and mice with constitutive activation of PKA show hyperphosphorylation of RyR2 and dilated cardiomyopathy [13].  Investigation of the downstream targets of PKA and how they affect cardiac function in aged RIIβ null mice will be a productive area of aging research.

## Deletion of the Cβ catalytic subunit of PKA results in delayed aging

We have studied PKA catalytic Cβ subunit null mutant mice to establish correlations with age-delaying benefits [18]. Female PKA Cβ null mice fed a high caloric diet (HCD) showed robust obesity resistance. The significant increase in body weight in wild type littermates was shown by quantitative magnetic resonance (QMR) imaging to be due to an increase in fat mass.  Generally, there was no difference in the amount of food consumed by either genotype.  When individual fat depots were weighed there was a sparing effect in visceral fat in both female and male PKA Cβ null mice consistent with observations indicating that accumulation of visceral fat is a high risk factor for age-related disease. Mutants of both genders also showed dramatic fat sparing effects in the liver, showing that PKA Cβ null mice are resistant to the hepatic steatosis-like condition associated with ingesting a high caloric diet. Blood glucose was elevated in wild type littermates, but not PKA Cβ null mice, as early as 4 weeks on the HCD.  A glucose tolerance test showed that PKA Cβ null mice on a HCD maintain their tolerance to glucose in contrast to wild type littermates. Hyperinsulinemia was seen as early as seven weeks into the HCD diet in wild type littermates, but not in PKA Cβ null mice. An insulin sensitivity test showed that PKA Cβ null mice do not develop insulin resistance associated with the high caloric diet as wild type littermates do.

**Figure 2.**
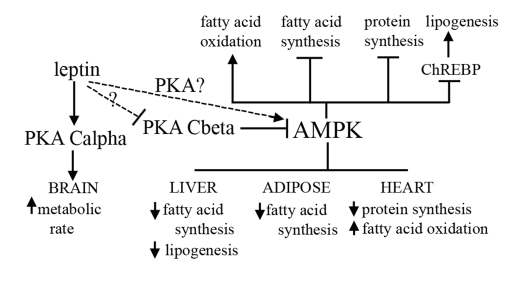
**Proposed mechanism for how the PKA Cβ deletion results in resistance to obesity, fatty liver, and heart disease.** Activation of AMPK is known to affect different aspects of lipid metabolism, and to play a role in protein synthesis.  PKA inhibits activity of AMPK, and we have shown that loss of Cβ results in decreased levels of ChREBP. Our model proposes that disruption of Cβ and concomitant increased AMPK activity leads to a decrease in fatty acid and protein synthesis and an increase in lipolysis and fatty acid oxidation in select tissues.  Leptin sensitivity caused by disruption of Cβ may also play a role in the observed increase in AMPK activity in our mutants.  A compensatory increase by Cα in the brain also results in an increase in overall energy expenditure.

In addition to being protected from diet-induced pathologies, PKA Cβ null mutant mice are protected from age-related problems such as weight gain and enlarged livers, as well as cardiac dysfunction and hypertrophy.  As with RIIβ, we have used echocardiography and doppler imaging to look at diastolic function and myocardial performance index, and have found superior Ea/Aa ratios and MPIs in the mutants compared to WT as early as 9 months of age, continuing up to 24 months of age.  By 24 months, we have observed other evidence of worsening diastolic function in WT mice, including significantly higher injection response times, reduced fractional shortening percentages, and enlarged left atria compared to mutants.  By end of life, we have found that WT mice have significantly larger hearts than littermates lacking PKA Cβ (manuscript in preparation).  The mechanisms for these observations are not known since PKA Cβ is detectable only at very low levels in cardiac tissue of the mouse.  We know that PKA Cβ is expressed in the liver and could help provide a correlation with cardiac protective effects since PKA has been shown to phosphorylate and inactivate AMPKα in order to regulate the activity of lipolytic enzymes such as hormone-sensitive lipase [19].  Increases in AMPKα  have been linked to fatty liver resistance, as well as a reduction in cardiac protein synthesis and delayed hypertrophy [20, 21].  Interestingly, we have data to show that Cβ null mice have increased levels of phosphorylated AMPK.  Transcription of the gene for carboyhydrate-response-element-binding protein ChREBP, a master regulator of lipid metabolism, is known to be AMPK-mediated, and we have found that levels of this protein are lower in livers of PKA Cβ disrupted mice. Increased fatty acid oxidation and lipolysis, and decreased fatty acid and protein synthesis through the AMPK pathway may be possible mechanisms by which PKA Cβ disruption leads to obesity resistance and healthy aging (Figure 2).

The high levels of Cβ expression in the brain, and the discrete neural expression of Cβ variants hints at a specific functional role in neuronal signaling. Disruption of Cβ causes a 26% decrease in basal PKA activity in the brain despite a reported compensatory increase in the amount of Cα protein [22]. While the catalytic subunits Cα and Cβ are 91% identical in amino acid sequence, their amino acid differences are highly conserved across species and they are thus believed to have unique functions [23]. Therefore, a shift from Cβ to Cα activity may still represent an increase in a particular type of PKA catalytic function.  Since our current studies suggest that PKA Cβ null mice are leptin sensitive, one possibility is an enhanced response to the activation of leptin-sensitive melanocortin receptors, resulting in increased energy expenditure compared to WT. The arcuate nucleus region of the hypothalamus contains leptin-responsive neurons that control feeding and energy expenditure through the activation of Gs-coupled melanocortin receptors.  These receptors are thought to decrease food intake and increase energy expenditure through stimulation of the cAMP pathway and activation of PKA [24]. The implications for aging are highly relevant since aging is known to be characterized by a decline in metabolic function, and is associated with resistance to the effects of leptin on the modulation of fat accumulation and distribution [25].  Interestingly, the AMPK pathway is also stimulated by leptin [26], suggesting another potential mechanism by which leptin sensitivity caused by deletion of PKA Cβ might lead to the obesity, fatty liver, and heart disease resistance phenotypes observed in our mice (Figure 2).

## PKA subunit genes are potential anti-aging targets

Since PKA is a major metabolic regulator of gene signaling, the human gene homologs are potential pharmacological targets for age-related conditions. Therefore, our studies in the mouse are directly applicable to advancing new knowledge in the treatment and prevention of diseases associated with progressive aging [27]. The heart would appear to be an excellent PKA inhibitory target since PKA null mutant mice are robustly protected from age-related cardiac decline.  Through the mouse models we have characterized, we hope to explore several possible mechanisms that may explain the positive health benefits of PKA inactivation or down-regulation. Studies on PKA subunit genes can be carried out and confirmed to more specially define the intervention targets and realistically predict biological outcomes in human clinical trials.
